# Effect of the Composition of Lanthanide Complexes on Their Luminescence Enhancement by Ag@SiO_2_ Core-Shell Nanoparticles

**DOI:** 10.3390/nano8020098

**Published:** 2018-02-09

**Authors:** Xiao-Jing Wang, Yan-Rong Qu, Yong-Liang Zhao, Hai-Bin Chu

**Affiliations:** College of Chemistry and Chemical Engineering, Inner Mongolia University, Huhhot 010021, China; 15248077022@163.com (X.-J.W.); quyanrong@126.com (Y.-R.Q.); hxzhaoyl@163.com (Y.-L.Z.)

**Keywords:** lanthanide complex, Ag@SiO_2_ nanoparticles, metal-enhanced luminescence, europium, terbium

## Abstract

Metal-enhanced luminescence of lanthanide complexes by noble metal nanoparticles has attracted much attention because of its high efficiency in improving the luminescent properties of lanthanide ions. Herein, nine kinds of europium and terbium complexes—RE(TPTZ)(ampca)_3_·3H_2_O, RE(TPTZ)(BA)_3_·3H_2_O, RE(phen)(ampca)_3_·3H_2_O, RE(phen)(PTA)_1.5_·3H_2_O (RE = Eu, Tb) and Eu(phen)(BA)_3_·3H_2_O (TPTZ = 2,4,6-tri(2-pyridyl)-s-triazine, ampca = 3-aminopyrazine-2-carboxylic acid, BA = benzoic acid, phen = 1,10-phenanthroline, PTA = phthalic acid)—have been synthesized. Meanwhile, seven kinds of core-shell Ag@SiO_2_ nanoparticles of two different core sizes (80–100 nm and 40–60 nm) and varied shell thicknesses (5, 12, 20, 30 and 40 nm) have been prepared. The combination of these nine types of lanthanide complexes and seven kinds of Ag@SiO_2_ nanoparticles provides an opportunity for a thorough investigation of the metal-enhanced luminescence effect. Luminescence spectra analysis showed that the luminescence enhancement factor not only depends on the size of the Ag@SiO_2_ nanoparticles, but also strongly relates to the composition of the lanthanide complexes. Terbium complexes typically possess higher enhancement factors than their corresponding europium complexes with the same ligands, which may result from better spectral overlap between the emission bands of Tb complexes and surface plasmon resonance (SPR) absorption bands of Ag@SiO_2_. For the complexes with the same lanthanide ion but varied ligands, the complexes with high enhancement factors are typically those with excitation wavelengths located nearby the SPR absorption bands of Ag@SiO_2_ nanoparticles. These findings suggest a combinatorial chemistry strategy is necessary to obtain an optimal metal-enhanced luminescence effect for lanthanide complexes.

## 1. Introduction

Luminescent lanthanide compounds have been widely used in various fields, such as fluorescence materials [1,2,3], electroluminescence devices [4,5], fluorescence probes and labels in biological systems [6,7,8,9], because they have the advantages of narrow emission bands, long fluorescence lifetimes and large Stokes shifts [10,11,12,13,14]. However, because of the parity rule, the f–f transition of lanthanide ions is forbidden, which leads to their weak luminescence intensities [1,2]. To enhance their luminescence intensities, one traditional strategy is to coordinate with various organic ligands. The ligands can sensitize the central lanthanide ions by ligand-to-metal energy transfer, the so-called “antenna effect” [15,16]. Typically, ternary lanthanide complexes utilizing both anion ligands and neutral organic ligands have shown superior luminescence properties. The aromatic anion ligands, such as benzoic acid (BA), terephthalic acid (PTA), and 3-aminopyrazine-2-carboxylic acid (ampca) can efficiently absorb light and transfer energy to the lanthanide ions [17,18]. Furthermore, the introduction of aromatic neutral ligands, including 1,10-phenanthroline (phen) and 2,4,6-tri(2-pyridyl)-s-triazine (TPTZ) can replace the solvent molecules coordinated with lanthanide ions to reduce the quenching effect [17,19].

In the past decade, another strategy based on metal-enhanced luminescence has been explored for the enhancement of luminescence properties of lanthanide complexes [20,21,22,23,24,25,26]. Metal-enhanced luminescence (MEL) is based on the localized surface plasmon resonance (SPR) effect of noble metal nanoparticles, especially gold or silver [27]. MEL occurs when fluorophores are positioned near-field from the metal nanoparticles. The actual mechanism of MEL is still debated at present [28]. The classical far-field fluorescence descriptions suggested a modification in the fluorophores’ intrinsic radiative decay rates [29]. In a new description, the excited fluorophores bring a mirror dipole in the nearby metal nanoparticle, which itself radiates the coupled quanta with high efficiency, leading to significantly enhanced luminescence [30]. The core-shell Ag@SiO_2_ nanoparticles have shown potential applications in many fields, such as optical biosensing, bioimaging, immunoassay, catalysts and antibacterial agents [20,23,25,31,32,33,34]. To achieve an optimized MEL effect on lanthanide complexes, many efforts have been paid to the size control of the nanoparticles and the distance adjustment between the particles and the lanthanide complexes [23]. For example, core-shell Ag@SiO_2_ nanoparticles with core size of tens of nanometer and shell thicknesses between 20 to 50 nm are frequently found to be efficacious for the luminescence enhancement of Eu and Tb complexes [31,35,36,37]. The SiO_2_ shell is important for avoiding the lanthanide complexes from having direct contact with the metal particles, which may lead to luminescence quench. Moreover, we have reported recently that the luminescence intensities of lanthanide complexes can also be tuned by controlling the ratios of the complexes and nanoparticles [18].

Furthermore, the luminescent properties of the lanthanide complexes depend strongly on their compositions, i.e., both the kind of lanthanide ions and the coordinated ligands. Herein, we synthesize nine kinds of Eu and Tb ternary complexes with both anion and neutral ligands, and prepare seven types of core-shell Ag@SiO_2_ nanoparticles of distinct size. By systematically comparing the luminescence enhancement effect of these complexes by these nanoparticles (9 × 7 = 63 combinations), the enhancement factors are found to not only rely on the size of the Ag@SiO_2_ nanoparticles, but also relate to the complex composition. The mechanism investigation indicates that the excitation enhancement and emission enhancement may lead to distinct enhancement factors for complexes with different lanthanide ions and organic ligands.

## 2. Experimental Section

### 2.1. Materials and Characterizations

The purities of terbium oxide and europium oxide were 99.99%. Silver nitrate (AgNO_3_), sodium citrate, tetraethyl orthosilicate (TEOS), ammonia (NH_3_·H_2_O), BA, PTA, phen, ampca, TPTZ, *N*,*N*-dimethylformamide (DMF) and other reagents were all of analytic grade and used as received.

Infrared spectra of the lathanide complexes and the ligands were recorded on a Nexus 670 FT-IR spectrophotometer (Nicolet, Madison, WI, USA) using KBr pellets. Molar conductivities were measured on a DDSJ-308A conductivity meter (Biocoteck, Ningbo, China) at room temperature using DMF as a solvent. Elemental analyses of C, H and N were performed by a Vario EL Cube elemental analysis instrument (Elementar, Hanau, Germany). Transmission electron microscopy (TEM, Tecnai F20, 200 kV, FEI, Hillsboro, OR, USA) was used to investigate the size and morphology of Ag@SiO_2_ nanoparticles. The UV-vis absorption spectra of the lathanide complexes and the core-shell Ag@SiO_2_ nanoparticles were recorded on a TU-1901 spectrophotometer (Beijing Purkinje, Beijing, China). The luminescence spectra of the lathanide complexes and the complex-doped Ag@SiO_2_ nanocomposites were recorded on the FLS-920 fluorescent spectrometer (Edinburgh Instruments, Livingston, UK) at room temperature.

### 2.2. The Preparation of Core-Shell Ag@SiO_2_ Nanoparticles

Two kinds of Ag nanoparticles, with particle sizes of 80–100 nm and 40–60 nm, respectively, were prepared by reducing AgNO_3_ with sodium citrate in water, as described in previous works [36,37]. The Ag nanoparticles were collected by centrifugation and finally, dispersed in ethanol. 

The methods for preparing Ag@SiO_2_ nanoparticles with different shell thicknesses were explored by controlling the amount of TEOS added. The typical process is as follows: 10 mL 40–60 nm Ag nanoparticle solution was added to a three round-bottom flask (250 mL), 30 mL ethanol and 0.2 mL sodium citrate solution was added under stirring at room temperature. After the pH value of the solution was adjusted to 9 with ammonia, 5, 10 or 15 mL of TEOS were added dropwise under vigorous stirring. The reaction lasted for 24 h. The core-shell Ag@SiO_2_ nanoparticles with shell thickness of 12, 30 or 40 nm were obtained by centrifugation. The other series of core-shell Ag@SiO_2_ nanoparticles with core sizes of 80–100 nm and shell thicknesses of 5, 12, 20 and 30 nm were prepared by varying the amounts of TEOS added, by 5, 7, 10 and 15 mL, respectively.

### 2.3. Synthesis of Lanthanide Complexes

The solution of EuCl_3_ was prepared by the reaction of Eu_2_O_3_ with HCl solution. The solution was heated to remove extra HCl, after which ethanol was added to dissolve the europium chloride. Preparation of TbCl_3_ ethanol solution is similar to that of EuCl_3_, except H_2_O_2_ was added. 

Typically, anionic ligands (3.0 mmol BA or ampca, or 1.5 mmol PTA), neutral ligands (1 mmol Phen or TPTZ) and 10 ml of anhydrous ethanol were added in the round bottom flask, and then the mixture was heated in a 60 °C water bath to obtain a clear solution. One mmol EuCl_3_ or TbCl_3_ was added into the above solution under stirring. The pH value of the solution was adjusted to about 6.4 with ammonia. After being heated for 3 h, the mixture was cooled to room temperature and left still overnight. Then the precipitates were filtered, washed with ethanol for several times and dried, to obtain the nine kinds of lanthanide complexes.

### 2.4. The Preparation of Complexes Doped Core-Shell Ag@SiO_2_ Nanocomposites

Two mL of the lanthanide complex in ethanol (1.0 × 10^−4^ mol·L^−1^) was put in a quartz cell and the luminescence spectrum was measured. Then, a small amount of Ag@SiO_2_ sol was added gradually until the strongest luminescence intensity was achieved. The total additional amount of Ag@SiO_2_ solution was 200 mL for each lanthanide complex.

## 3. Results and Discussions

### 3.1. Characterizations of the Core-Shell Ag@SiO_2_ Nanoparticles

**Figure 1:**
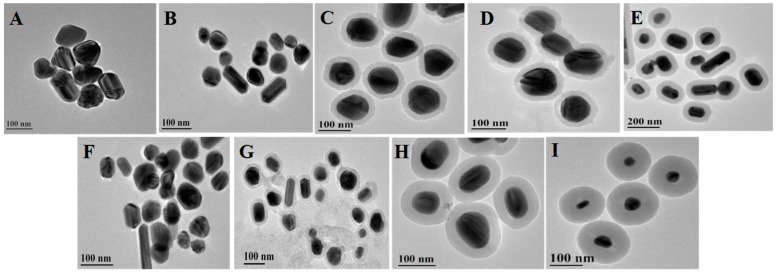
TEM images of Ag nanoparticles (**A**,**F**) and core-shell Ag@SiO_2_ nanoparticles (**B**–**E**,**G**–**I**). The Ag core sizes are 80–100 nm in (**A**–**E**) and 40–60 nm in (**F**–**I**). The silica shell thickness of (**B**–**E**) are 5, 12, 20, 30 nm, and (**G**–**I**) are 12, 30, 40 nm, respectively.

**Figure 2:**
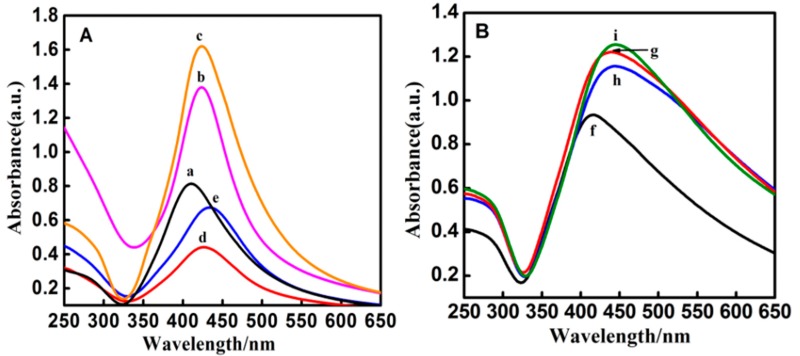
(**A**) UV-vis absorption spectra of Ag@SiO_2_ nanoparticles with core sizes of 80–100 nm (a–e) and SiO_2_ thicknesses of 5 nm (b), 12 nm (c), 20 nm (d) and 30 nm (e); (**B**) UV-vis absorption spectra of Ag@SiO_2_ nanoparticles with core sizes of 40–60 nm (f–i) and SiO_2_ thicknesses of 12 nm (g), 30 nm (h) and 40 nm (i).

Figure 1 shows typical TEM images of two kinds of silver nanoparticles (A and F) and seven kinds of core-shell Ag@SiO_2_ nanoparticles (B–E and G–I) with different shell thickness. The shell thickness was controlled carefully by the concentration of TEOS. The diameters of silver cores in Figure 1A–E are all about 80–100 nm, while the silica shell thicknesses of the Ag@SiO_2_ nanoparticles in Figure 1B–E are 5, 12, 20 and 30 nm, respectively. The diameters of the silver cores in Figure 1F–I are about 40–60 nm and the silica shell thicknesses of the Ag@SiO_2_ nanoparticles in Figure 1G–I are 12, 30 and 40 nm, respectively. The particle size and the surrounding medium will affect the surface plasmon resonance absorption of Ag@SiO_2_ nanoparticles in the visible wavelength region [38,39]. As shown in Figure 2, the UV-vis absorption bands of silver nanoparticles red-shift when they are coated with silica, which is consistent with Mie’s theory [24,40,41]. The absorption peak locates at 407 nm for the silver particles of 80–100 nm. After coating with silica of different thicknesses, the absorption peaks of Ag@SiO_2_ nanoparticles red-shift to 422, 423, 425 and 433 nm for the thicknesses of 5, 12, 20 and 30 nm, respectively. The 40–60 nm silver nanoparticles exhibit an absorption peak around 412 nm, which red-shifts to 426, 434 and 438 nm after coating a silica shell of 12, 30 and 40 nm, respectively.

### 3.2. Characterizations of Lanthanide Complexes

The data from the elemental analyses (C, H, N) and molar conductivities of the complexes are listed in Table 1. The results indicate that the compositions of the nine complexes are RE(phen)(ampca)_3_·3H_2_O, RE(TPTZ)(BA)_3_·3H_2_O, RE(TPTZ)(ampca)_3_·3H_2_O, RE(phen)(PTA)_1.5_·3H_2_O (RE = Tb and Eu) and Eu(phen)(BA)_3_·3H_2_O, respectively. The molar conductivity values of these complexes are in the range of 11.2–18.3 S·cm^2^·mol^−1^, which indicates that the lanthanide complexes are non-electrolytes [42,43].

The UV-vis absorption spectra of the complexes and the ligands were determined using the mixture of DMF and ethanol (*v*/*v* = 1/25) as a solvent and reference. Because the UV-vis absorption peak positions of terbium complexes and europium complexes are similar, so only the UV-vis absorption data of the europium complexes and ligands are listed in Table S1. As shown in Figure S1, the ligands TPTZ and BA exhibit strong absorption bands around 282 and 226 nm, respectively. However, the BA absorption band disappeared in the complex Eu(TPTZ)(BA)_3_·3H_2_O, and a new band around 246 nm occurred. Meanwhile, the TPTZ absorption peak moved to 283 nm. These shifts suggested the formation of the complexes. Moreover, the absorption peaks of the ligands—ampca, phen and PTA—appeared at 350, 263 and 230 nm, but in the complexes Eu(TPTZ)(ampca)_3_·3H_2_O, Eu(phen)(ampca)_3_·3H_2_O, Eu(phen)(BA)_3_·3H_2_O and Eu(phen) (PTA)_1.5_·3H_2_O, the absorption peaks appeared at 364, 356, 289 and 264 nm respectively. These changes also indicate that the ligands have coordinated with Eu^3+^. 

**Figure 3:**
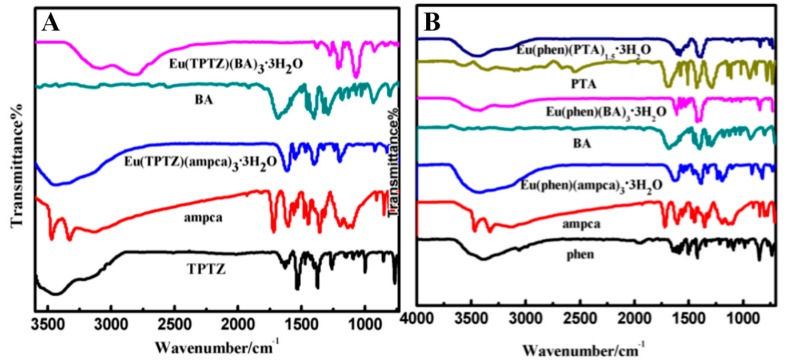
Infrared spectra of the Eu complexes and related ligands. (**A**) Eu(TPTZ)(BA)_3_·3H_2_O, Eu(TPTZ)(amcpa)_3_·3H_2_O, BA, ampca and TPTZ; (**B**) Eu(phen)(PTA)_1.5_·3H_2_O, Eu(phen)(BA)_3_·3H_2_O, Eu(phen)(ampca)_3_·3H_2_O, BA, ampca and phen.

Since the Infrared spectra of the terbium complexes and the europium complexes are similar, only the IR spectra of the ligands and the europium complexes are given in Figure 3. Comparing the spectra of ligands with those of the complexes, it can be seen that the characteristic absorption peaks of the ligands have shifted in the complexes, which indicates the europium ions may have coordinated with the ligands. For example, the spectrum of TPTZ shows the absorption bands around 1374 and 1000 cm^−1^, which could be ascribed to the central ring breathing vibration and pyridine ring bending vibration [44]. These two bands move to around 1400 and 1007 cm^−1^ in the complexes Eu(TPTZ)(ampca)_3_·3H_2_O and Eu(TPTZ)(BA)_3_·3H_2_O, which suggests that the central ring and pyridine ring of TPTZ have been involved in the coordination of the europium complexes. For the ligand ampca, the vibration band of carbonyl group at 1720 cm^−1^ disappears in the complexes, Eu(TPTZ)(ampca)_3_·3H_2_O and Eu(phen)(ampca)_3_·3H_2_O, and two new bands appear at 1605 cm^−1^ and 1355 cm^−1^, which can be assigned to the asymmetric and symmetric stretching vibration of carboxyl [45,46]. These changes indicate that the carboxyl of ampca has coordinated with Eu^3+^ ions in the complexes. The stretching vibration band of carbonyl group in BA at 1686 cm^−1^ disappears in the complexes, Eu(phen)(BA)_3_·3H_2_O and Eu(TPTZ)(BA)_3_·3H_2_O, but at 1544 and 1491 cm^−1^ the anti-symmetric and symmetric stretching vibration peaks of carboxyl emerge. These changes also suggest that the ligand BA has coordinated with Eu^3+^ ions in the complexes. The spectra changes of the ligand phen are similar in the complexes, Eu(phen)(ampca)_3_·3H_2_O, Eu(phen)(BA)_3_·3H_2_O and Eu(phen)(PTA)_1.5_·3H_2_O. The C=N stretching vibration absorption band of phen moves from 1586 to 1551 cm^−1^ in the complexes, which indicates that coordinate bonds have formed between the europium ions and phen [47]. For the complex Eu(phen)(PTA)_1.5_·3H_2_O, the stretching vibration peak of the carbonyl group in PTA at 1689 cm^−1^ disappears after coordinated with the europium ions and new bands at 1610 cm^−1^ and 1518 cm^−1^ appeared in the complex, which can be ascribed to the anti-symmetric and symmetric stretching vibration of carboxyl group, indicating that the europium ions have coordinated with PTA [48].

### 3.3. Luminescence Enhancement of the Lanthanide Complexes by Ag@SiO_2_ Nanoparticles

**Figure 4:**
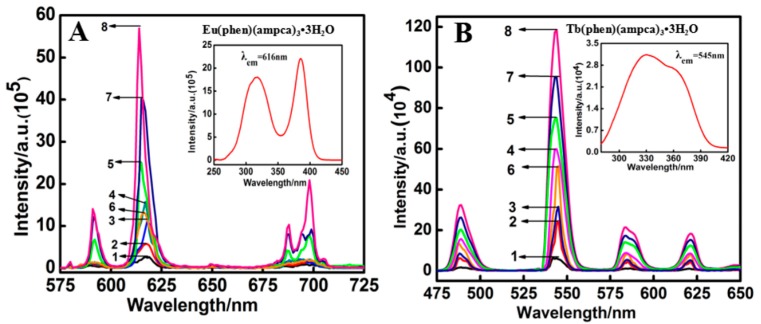
Luminescent emission spectra of Eu(phen)(ampca)_3_·3H_2_O (**A**) and Tb(phen)(ampca)_3_·3H_2_O (**B**) before and after the addition of varied Ag@SiO_2_ nanoparticles. The insets show the corresponding excitation spectra of the complexes. Sample 1 is the pure complex. Samples 2–5 represent the complexes added with the Ag@SiO_2_ nanoparticles with core sizes of 80–100 nm and shell thicknesses of 5, 12, 20 and 30 nm. Samples 6–8 represent the complexes added with the Ag@SiO_2_ nanoparticles with core sizes of 40–60 nm and shell thicknesses of 12, 30 and 40 nm, respectively.

The luminescence spectra of the europium and terbium complexes before and after adding core-shell Ag@SiO_2_ nanoparticles were obtained in ethanol solution. Both of the excitation and emission slit widths were 3 nm for all measurements. The excitation spectra were obtained by monitoring the emission wavelengths at 617 and 543 nm, for europium and terbium complexes, respectively. Then the emission spectra were determined at the most efficacious excitation wavelengths for each complex. As shown in Figure 4A and Figure S2, the five europium complexes all exhibit a typical Eu^3+^ emission pattern with a maximum emission peak around 617 nm (^5^D_0_→^7^F_2_) and several other peaks around 580, 590, 650 and 697 nm, respectively. Figure 4B and Figure S3 show that the four terbium complexes all possess a typical Tb^3+^ emission pattern with a maximum emission peak around 543 nm (^5^D_4_→^7^F_5_) and several other peaks around 489, 585 and 621 nm, respectively. All the peak positions do not shift obviously after adding Ag@SiO_2_ nanoparticles. Table 2 lists the luminescence emission intensities of the europium (^5^D_0_→^7^F_2_ transitions) and terbium complexes (^5^D_4_→^7^F_4_ transitions), both before and after the introduction of Ag@SiO_2_ nanoparticles of varied size. In the following discussion, Sample 1 stands for the pure rare earth complex, Samples 2–5 mean the nanocomposites after adding Ag@SiO_2_ nanoparticles with core sizes of 80–100 nm and shell thicknesses of 5, 12, 20, 30 nm, respectively, and Samples 6–8 denote the nanocomposites containing Ag@SiO_2_ nanoparticles with core sizes of 40–60 nm and shell thicknesses of 12, 30 and 40 nm, respectively. It can be seen that the emission intensities of the europium complexes (1.7–2.9 × 10^5^ a.u.) are typically stronger than those of the terbium complexes (0.60–0.64 × 10^5^ a.u.). The sensitization effect of the lanthanide ions by organic ligands is related to energy gap between the triplet energy level of ligands and lowest excited states energy of the lanthanide ions. It is believed that the intramolecular ligand-to-metal energy transfer is effective if the energy gap is about 2000–5000 cm^−1^ [49]. The triplet energy levels of the ligands, BA (23,800 cm^−1^), PTA (21560 cm^−1^) and ampca, are high enough for effective ligand-to-metal transfer processes in the complexes. Besides, the triplet energy levels of phen (20,850 cm^−1^) and TPTZ (21,277 cm^−1^) are higher than the ^5^D_0_ level (17,250 cm^−1^) of Eu^3+^ [50]. The energy gaps of the triplet energy levels of phen and TPTZ, with the lowest excited energy of the Eu^3+^ ion, are about 3600 and 4300 cm^−1^ respectively. Therefore, the anion and neutral ligands can transfer their absorbed light energy to Eu^3+^ ions effectively, and the europium complexes exhibit superior luminescence properties. For the terbium complexes, because the ^5^D_4_ level of Tb^3+^ is around 20,430 cm^−1^, the anionic ligands (BA, PTA and ampca) can still transfer the absorbed energy to Tb^3+^. However, the triplet energy levels of TPTZ and phen are almost resonant with ^5^D_4_ of Tb^3+^ [51,52]. Thus, back transfer would reduce the luminescence intensities of terbium complexes.

As shown in Figure 4 and Figures S2 and S3, the luminescence emission intensities of the lanthanide complexes are obviously enhanced after adding the Ag@SiO_2_ nanoparticles. It can be seen from Figure S4 that the four transitions of the terbium complexes (^5^D_4_→^7^F_6_, ^5^D_4_→^7^F_5_, ^5^D_4_→^7^F_4_ and ^5^D_4_→^7^F_3_) obtain similar enhancement factors with the same kind of Ag@SiO_2_. While the five transitions (^5^D_0_→^7^F_0_, ^5^D_0_→^7^F_1_, ^5^D_0_→^7^F_2_, ^5^D_0_→^7^F_3_ and ^5^D_0_→^7^F_4_) of the europium complexes possess quite different enhancement factors with the same kind of Ag@SiO_2_ (Figure S5), and the enhancement factor of ^5^D_0_→^7^F_2_ is the largest among them. The difference in enhancement factors has also been checked by superimposing the emission spectra in a normalized way (Figure S6). The difference cannot be well explained at present. We only discuss the enhancement factors of the strongest emission peaks, as follows. For example, the luminescence enhancement factors of the complex Eu(phen)(ampca)_3_·3H_2_O at ^5^D_0_→^7^F_2_ transition reached 2.14, 4.23, 5.91, 9.32, 4.96, 14.96 and 21.40 times, with the seven kinds of Ag@SiO_2_ nanoparticles (Table 2, Samples 2–8), respectively. The highest enhancement factor was reached by Ag@SiO_2_ nanoparticles (Sample 8) with core diameters of 40–60 nm and a shell thickness of 40 nm. For the other four europium complexes—Eu(phen)(BA)_3_·3H_2_O, Eu(phen)(PTA)_1.5_·3H_2_O, Eu(TPTZ)(BA)_3_·3H_2_O and Eu(TPTZ)(amcpa)_3_·3H_2_O—the maximum enhancement factor was also reached by the same Ag@SiO_2_ nanoparticles. Their enhancement factors at ^5^D_0_→^7^F_2_ transitions are 14.7, 15.99, 12.47 and 16.91 (Figure S2 and Table 2), respectively. The enhancement factor strongly depends on the shell thickness and core diameter. For the Ag@SiO_2_ nanoparticles with core diameters of 80–100 nm, the enhancement factors for the same complex increase with the order of samples: 2, 3, 4, 5 (shell thickness of 5, 12, 20, 30 nm). In addition, for the Ag@SiO_2_ nanoparticles with core diameters of 40–60 nm, the enhancement factors for the same complex increase with the order of samples: 6, 7, 8 (shell thickness of 12, 30, 40 nm). When the SiO_2_ shell is too thin, the distance between the europium complexes and silver core is so close that it may lead to non-radiative energy transfer from the europium complexes to the silver core. Thus, the emission intensities of the complex-doped Ag@SiO_2_ nanocomposites with thinner silica shells are weaker than those of nanocomposites with thicker silica shells, in the range of 5–40 nm [35,36]. The core diameter of the nanoparticle also plays key roles in the enhancement effect. For example, Samples 3 and 6 have Ag@SiO_2_ nanoparticles with the same shell thickness of 12 nm, but with different core sizes (80–100 nm and 40–60 nm). The enhancement factors of Sample 6 are much higher than those of Sample 3 for all the nine lanthanide complexes (Table 2). The same trend can be found in Samples 5 and 7 with the same shell thickness of 30 nm, which is also similar to our previous report [18].

**Figure 5:**
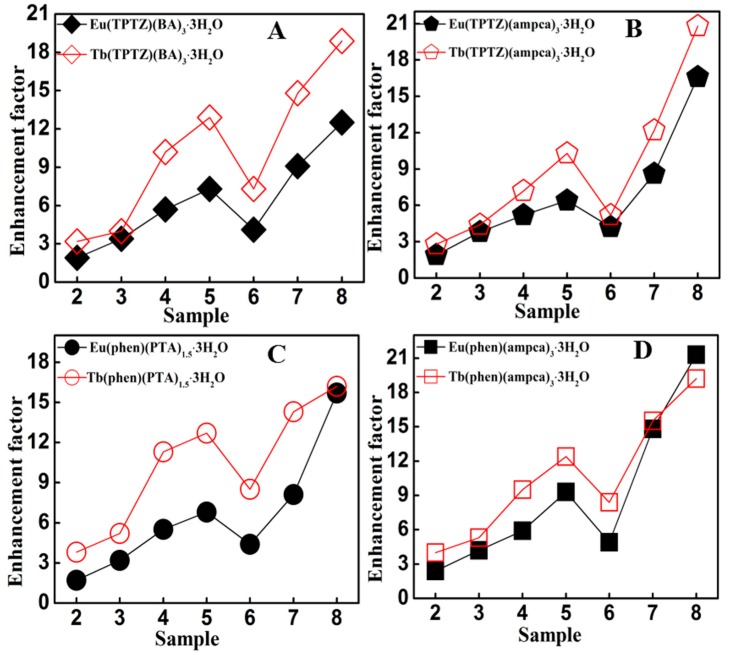
Comparison of the luminescence enhancement factors between Tb and Eu complexes. (**A**) Eu(TPTZ)(BA)_3_·3H_2_O and Tb(TPTZ)(BA)_3_·3H_2_O; (**B**) Eu(TPTZ)(amcpa)_3_·3H_2_O and Tb(TPTZ)(amcpa)_3_·3H_2_O; (**C**) Eu(phen)(PTA)_1.5_·3H_2_O and Tb(phen)(PTA)_1.5_·3H_2_O; (**D**) Eu(phen)(ampca)_3_·3H_2_O and Tb(phen)(ampca)_3_·3H_2_O.

The luminescence properties of lanthanide complexes strongly depend on their compositions, including the lanthanide ions and the ligands, both of which may affect metal-enhanced luminescence. First, metal-enhanced luminescence of Tb and Eu complexes with the same ligands was systematically compared. As shown in Figure 5A and Table 2, the enhancement factors of the terbium complex Tb(TPTZ)(BA)_3_·3H_2_O are 3.2, 4.0, 10.2, 13.0, 7.4, 14.8 and 19.2, respectively, in the composites with the seven kinds of Ag@SiO_2_. These enhancement factors are much higher than those of the corresponding europium complex Eu(TPTZ)(BA)_3_·3H_2_O (2.4, 3.4, 5.7, 7.3, 4.0, 9.1 and 12.5). The same phenomena can be found in Tb(TPTZ)(ampca)_3_·3H_2_O vs. Eu(TPTZ)(ampca)_3_·3H_2_O (Figure 5B and Table 2), Tb(phen)(PTA)_1.5_·3H_2_O vs. Eu(phen)(PTA)_1.5_·3H_2_O (Figure 5C and Table 2) and Tb(phen)(ampca)_3_·3H_2_O vs. Eu(phen)(ampca)_3_·3H_2_O (Figure 5D and Table 2). Therefore, it can be concluded that the Tb complexes typically exhibit higher enhancement factors than Eu complexes with identical ligands and Ag@SiO_2_ nanoparticles.

**Figure 6:**
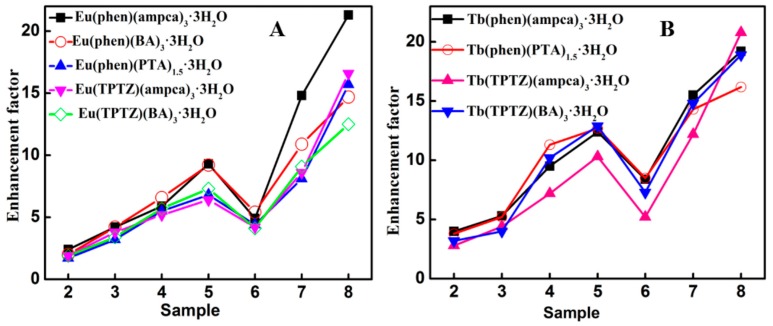
Comparison of the luminescence enhancement factors among the lanthanide complexes with different ligands. (**A**) Eu complexes; (**B**) Tb complexes.

Second, the ligand also plays an important role in the metal-enhanced luminescence of lanthanide complexes. Figure 6A compares five kinds of Eu complexes with varied ligands. The highest enhancement factor of 21.4 is reached by Eu(phen)(ampca)_3_·3H_2_O in Sample 8. The corresponding enhancement factors of Eu(TPTZ)(ampca)_3_·3H_2_O, Eu(phen)(PTA)_1.5_·3H_2_O, Eu(phen)(BA)_3_·3H_2_O and Eu(TPTZ)(BA)_3_·3H_2_O are 16.9, 16.0, 14.6 and 12.5, respectively, with the same Ag@SiO_2_ nanoparticles. Note that the excitation wavelengths of these five complexes are 386, 366, 310, 291 and 276 nm, respectively. It seems that the enhancement factors would be large when the excitation wavelengths of the complexes locate nearby the SPR absorption peaks of the Ag@SiO_2_ nanoparticles (438 nm). Moreover, in Sample 8 (Figure 6B), the three terbium complexes, Tb(phen)(ampca)_3_·3H_2_O, Tb(TPTZ)(ampca)_3_·3H_2_O and Tb(TPTZ)(BA)_3_·3H_2_O, with excitation wavelengths at 330, 306 and 312 nm, also show higher enhancement factors (19.0, 20.6 and 19.2) than Tb(phen)(PTA)_1.5_·3H_2_O (16.1) whose excitation wavelength is much shorter at 276 nm.

The metal-enhanced luminescence of the lanthanide complex is considered to result from the following aspects [18,36,53]: (i) excitation enhancement due to increased light absorption; (ii) emission enhancement due to increased light absorption and radiative decay; (iii) quenching reduction due to decreased nonradiative decay. The varied enhancement factors of Tb and Eu complexes may be mainly attributed to the emission enhancement. Compared with the emission bands of Eu complexes (^5^D_0_→^7^F_2_ around 617 nm), the strongest emission bands of Tb complexes (^5^D_4_→^7^F_4_ around 543 nm) are much closer to the SPR absorption bands of Ag@SiO_2_ nanoparticles (peaks at 422–438 nm). Theoretical calculations have predicted that the highest fluorescence would come from fluorophores with emission peaks slightly lower energy than the SPR scattering peak [30]. Therefore, the luminescence enhancement factors of the Tb complexes are typically higher than the corresponding Eu complexes. Meanwhile, the ligand effect on the enhancement factors of the lanthanide complexes may arise from the varied excitation enhancement at different excitation wavelengths. The excitation wavelength of the lanthanide complexes can be varied over a wide range, from UV to visible light, with various ligands. The SPR absorption bands of Ag@SiO_2_ nanoparticles lie in the wavelength range of 300–500 nm, which could overlap with the excitation spectra of the lanthanide complexes. For example, the excitation wavelength of complex Eu(phen)(ampca)_3_·3H_2_O is 386 nm, lying in the middle of the SPR absorption band of Ag@SiO_2_ nanoparticles. As a result, the excitation of the complex could be enhanced enormously by the SPR absorption of Ag@SiO_2_ nanoparticles. Thus, the luminescence enhancement factor of Eu(phen)(ampca)_3_·3H_2_O is the highest among the five Eu complexes.

## 4. Conclusions

In summary, nine kinds of europium and terbium complexes have been synthesized. The compositions of them were confirmed to be RE(phen)(ampca)_3_·3H_2_O, RE(TPTZ)(BA)_3_·3H_2_O, RE(TPTZ)(ampca)_3_·3H_2_O, RE(phen)(PTA)_1.5_·3H_2_O (RE = Tb and Eu) and Eu(phen)(BA)_3_·3H_2_O by elemental analysis, molar conductivity measurement, UV–vis absorption spectra and IR spectra. Then, luminescence emissions of these complexes were enhanced by two series of Ag@SiO_2_ nanoparticles of varied sizes. Though the luminescence emission intensities of the europium complexes were stronger than the terbium complexes of the same ligands, the terbium complexes typically exhibited higher luminescence enhancement factors than the europium complexes after the introduction of Ag@SiO_2_ nanoparticles. Meanwhile, for the complex with the same lanthanide ions but different ligands, the closer excitation wavelength to the SPR absorption bands of Ag@SiO_2_, the stronger luminescence enhancement extent was. The highest luminescence enhancement fator of 21.4 was reached by the Eu(phen)(ampca)_3_·3H_2_O complex, whose excitation wavelength (386 nm) lies in the middle of the SPR absorption band of Ag@SiO_2_ nanoparticles. Therefore, if we can set up two databases, one consisting of the luminescence excitation and emission spectra of the lanthanide complexes with various compositions, and the other being composed of the SPR absorption spectra of the metal nanoparticles of distinct size, structure and compositions, optimization of the metal-enhanced luminescence of a certain complex with a combinatorial chemistry method would be realized with high efficiency.

## Figures and Tables

**Table 1:** Data of elemental analysis and molar conductivities of the complexes.

Complex	C(%)	H(%)	N(%)	Conductivity (S·cm^2^·mol^−1^)
Eu(phen)(ampca)_3_·3H_2_O	40.03(40.48)	3.56(3.50)	18.92(19.24)	11.2
Eu(TPTZ)(BA)_3_·3H_2_O	53.46(53.18)	4.22(4.07)	9.98(9.55)	13.1
Eu(TPTZ)(ampca)_3_·3H_2_O	42.88(42.46)	3.67(3.22)	22.90(22.52)	12.6
Eu(phen)(BA)_3_·3H_2_O	52.47(52.62)	4.47(4.52)	3.79(3.72)	13.2
Eu(phen)(PTA)_1.5_·3H_2_O	45.47(45.33)	3.46(3.17)	3.97(4.41)	14.2
Tb(phen)(ampca)_3_·3H_2_O	40.57(40.14)	3.59(3.25)	19.47(19.08)	14.2
Tb(TPTZ)(BA)_3_·3H_2_O	52.07(52.49)	4.03(4.04)	9.78(9.42)	18.3
Tb(TPTZ)(ampca)_3_·3H_2_O	42.57(42.17)	3.59(3.22)	22.70(22.37)	17.4
Tb(phen)(PTA)_1.5_·3H_2_O	45.37(45.14)	3.47(3.73)	4.48(4.38)	15.9

Note: the values in brackets are theoretical values. phen, 1,10-phenanthroline; ampca, 3-aminopyrazine-2-carboxylic acid; BA, benzoic acid; PTA, phthalic acid; TPTZ, 2,4,6-tri(2-pyridyl)-s-triazine.

**Table 2:** Luminescence emission data of the complexes and complex-doped Ag@SiO_2_ nanocomposites at the strongest emissions (europium complex at ^5^D_0_→^7^F_2_ transitions, terbium complex at ^5^D_4_→^7^F_4_ transitions).

Complex	λ_ex_ (nm)	Emission Intensity (a.u.) (10^5^)							
		1	2	3	4	5	6	7	8
Eu(phen)(ampca)_3_·3H_2_O	386	2.7	5.7(2.1)	11.4(4.2)	15.8(5.9)	25.0(9.3)	13.2(4.9)	39.9(14.8)	57.6(21.3)
Eu(phen)(BA)_3_·3H_2_O	291	1.7	3.4(2.0)	7.1(4.2)	11.3(6.6)	15.6(9.2)	9.1(5.4)	18.5(10.9)	25.0(14.7)
Eu(phen)(PTA)_1.5_·3H_2_O	276	2.3	3.8(1.7)	7.3(3.2)	12.6(5.5)	15.6(6.8)	10.0(4.4)	18.6(8.1)	36.1(15.7)
Eu(TPTZ)(ampca)_3_·3H_2_O	366	2.9	5.4(1.9)	11.1(3.8)	15.0(5.2)	18.7(6.4)	12.1(4.2)	24.8(8.6)	48.2(16.6)
Eu(TPTZ)(BA)_3_·3H_2_O	310	2.0	3.8(1.9)	6.8(3.4)	11.3(5.7)	14.6(7.3)	8.1(4.1)	18.2(9.1)	25.0(12.5)
Tb(phen)(ampca)_3_·3H_2_O	330	0.62	2.5(4.0)	3.3(5.3)	5.9(9.5)	7.7(12.4)	5.2(8.4)	9.6(15.5)	11.9(19.2)
Tb(phen)(PTA)_1.5_·3H_2_O	306	0.60	2.3(3.8)	3.1(5.2)	6.8(11.3)	7.6(12.7)	5.1(8.5)	8.6(14.3)	9.7(16.2)
Tb(TPTZ)(ampca)_3_·3H_2_O	273	0.64	1.8(2.8)	2.8(4.4)	4.6(7.2)	6.6(10.3)	3.3(5.2)	7.8(12.2)	13.3(20.8)
Tb(TPTZ)(BA)_3_·3H_2_O	312	0.63	2.0(3.2)	2.5(4.0)	6.4(10.2)	8.1(12.9)	4.6(7.3)	9.3(14.8)	11.9(18.9)

Note: The data in brackets are the luminous enhancement factors.

## Supplementary Materials

The following are available online at http://www.mdpi.com/2079-4991/8/2/98/s1.
