# Supplementary material for: Effect of the Composition of Lanthanide Complexes on Their Luminescence Enhancement by Ag@SiO2 Core-Shell Nanoparticles
Source: Nanomaterials (Basel). 2018 Feb 9;8(2):98. doi: 10.3390/nano8020098 (PMC5853729; doi:10.3390/nano8020098)
Supplement: Supplementary file 1 [file nanomaterials-08-00098-s001.pdf]

Supplementary Materials

Effect of the Composition of Lanthanide Complexes on Their Luminescence Enhancement by Ag@SiO_2 Core-Shell Nanoparticles

Xiao-Jing Wang, Yan-Rong Qu, Yong-Liang Zhao and Hai-Bin Chu *

College of Chemistry and Chemical Engineering, Inner Mongolia University, Huhhot 010021, China; 15248077022@163.com (X.-J.W.); quyanrong@126.com (Y.-R.Q.); hxzhaoyl@163.com (Y.-L.Z.)
* Correspondence: chuhb@imu.edu.cn; Tel.:+86-471-499-2692

1. UV-vis absorption data of ligands and complexes

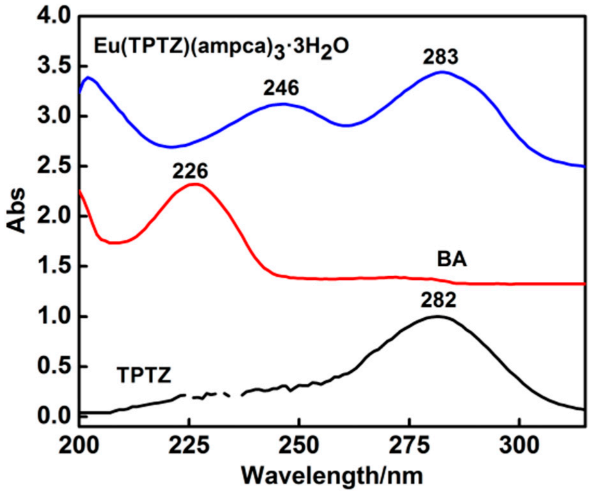

Fig S1. UV-vis absorption spectra of Eu(TPTZ)(BA)$_3$·3H_2O and ligands.

Table S1. UV-vis absorption peak positions of complexes and ligands.

Ligands and complexes	Peak position (nm)
TPTZ	282
BA	226
phen	263
PTA	239
ampca	350
Eu(phen)(ampca)$_3$·3H_2O	356
Eu(TPTZ)(BA)$_3$·3H_2O	283
Eu(TPTZ)(ampca)$_3$·3H_2O	364
Eu(phen)(BA)$_3$·3H_2O	289
Eu(phen)(PTA)$_{1.5}$·3H_2O	264

2. Luminescence emission spectra of the complexes

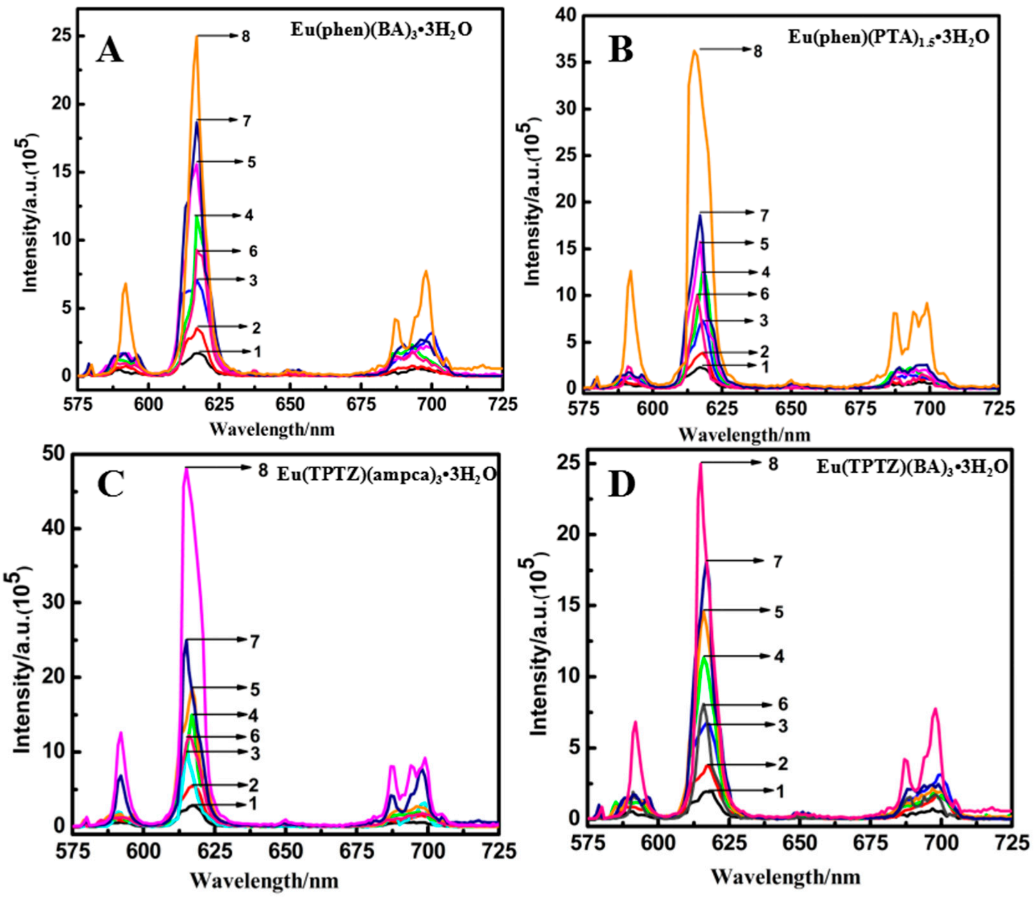

Fig S2. Luminescence emission spectra of Eu complexes with Ag@SiO_2 nanoparticles. (**A**) Eu(phen)$(BA)_3$·3H_2O; (**B**) Eu(phen)$(PTA)_{1.5}$·3H_2O; (**C**) Eu(TPTZ)$(amcpa)_3$·3H_2O; (**D**) Eu(TPTZ)$(BA)_3$·3H_2O. Sample 1 is the pure complex. 2~5 represent the complex added with the Ag@SiO_2 nanoparticles with core size of 80–100 nm and shell thickness of 5, 12, 20, 30 nm. 6~8 represent the complex added with the Ag@SiO_2 nanoparticles with core size of 40–60 nm and shell thickness of 12, 30 and 40 nm, respectively.

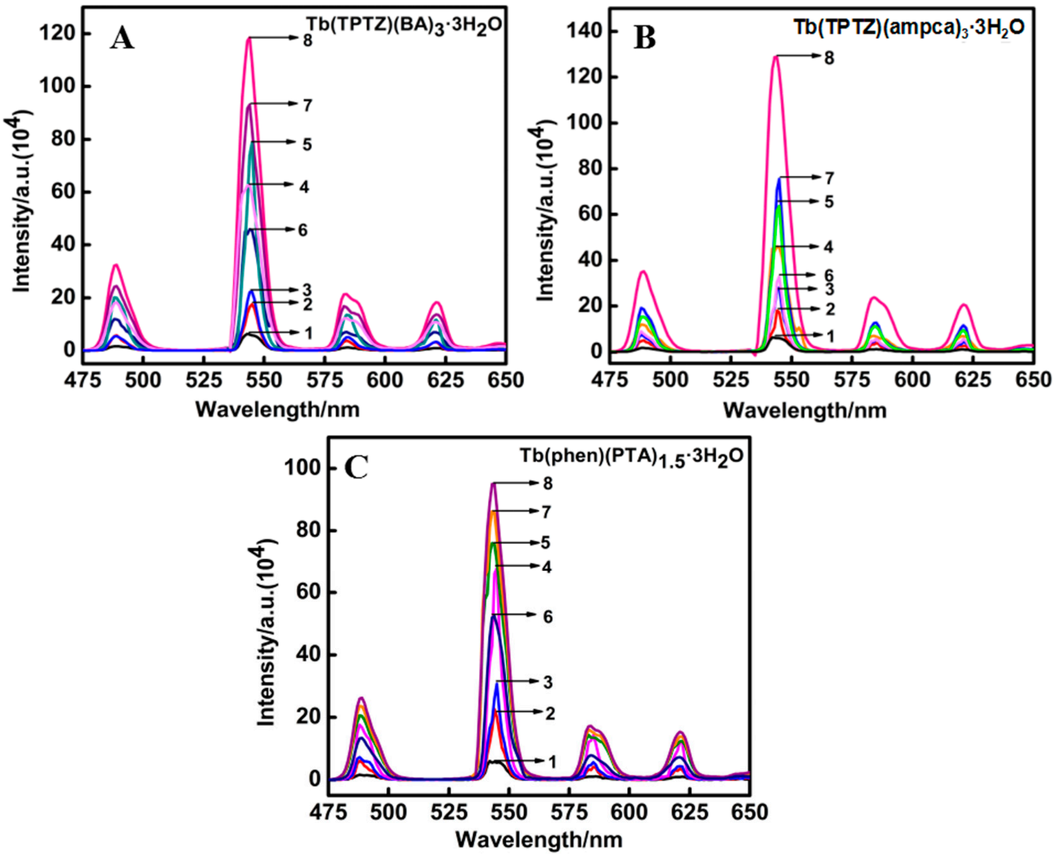

Fig S3. Luminescence emission spectra of Tb complexes with Ag@SiO_2 nanoparticles. (**A**) Tb(TPTZ)$(BA)_3$·$3H_2O$; (**B**) Tb(TPTZ)$(amcpa)_3$·$3H_2O$; (**C**) Tb(phen)$(PTA)_{1.5}$·$3H_2O$. Sample 1 is the pure complex. 2~5 represent the complex added with the Ag@SiO_2 nanoparticles with core size of 80–100 nm and shell thickness of 5, 12, 20, 30 nm. 6~8 represent the complex added with the Ag@SiO_2 nanoparticles with core size of 40-60 nm and shell thickness of 12, 30 and 40 nm, respectively.

3. Luminescent Enhancement factor of the complexes

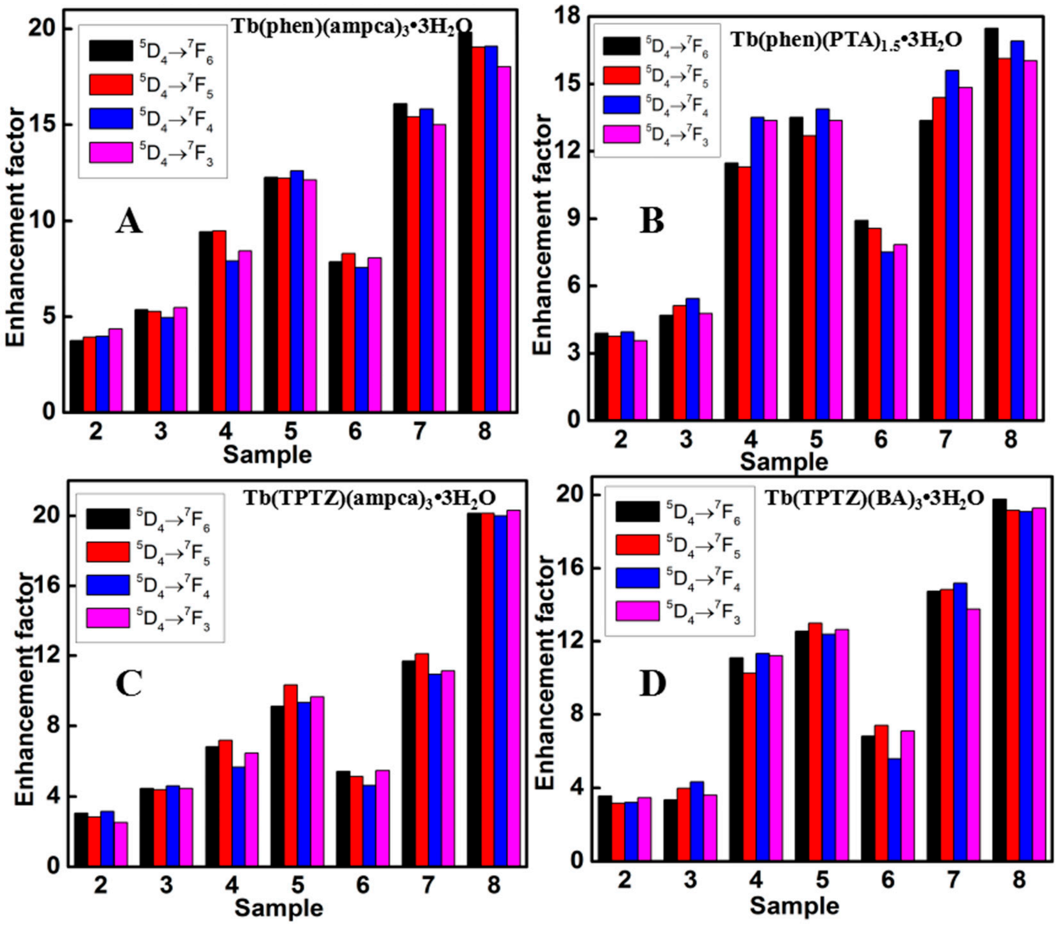

Fig S4. Luminescence enhancement factor of Tb complexes with Ag@SiO$_2$ nanoparticles. (**A**) Tb(phen)(ampca)$_3$·3H$_2$O; (**B**) Tb(phen)(PTA)$_{1.5}$·3H$_2$O; (**C**) Tb(TPTZ)(amcpa)$_3$·3H$_2$O; (**D**) Tb(TPTZ)(BA)$_3$·3H$_2$O. Samples 2~5 represent the complex added with the Ag@SiO$_2$ nanoparticles with core size of 80–100 nm and shell thickness of 5, 12, 20, 30 nm. 6~8 represent the complex added with the Ag@SiO$_2$ nanoparticles with core size of 40–60 nm and shell thickness of 12, 30 and 40 nm, respectively.

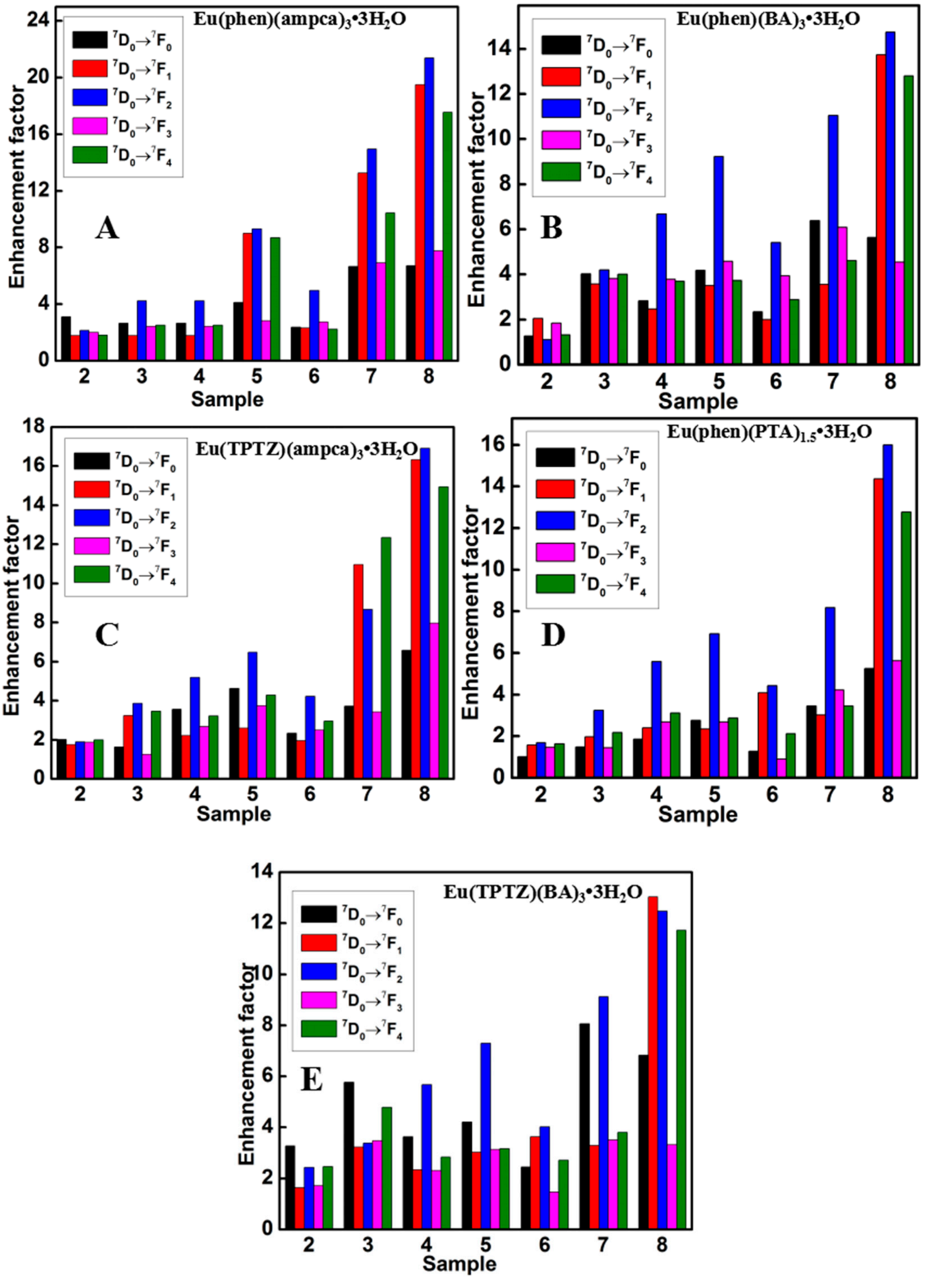

Fig S5. Luminescence enhancement factor of Eu complexes with Ag@SiO$_2$ nanoparticles. (**A**) Eu(phen)(ampca)$_3$·3H$_2$O; (**B**) Eu(phen)(BA)$_3$·3H$_2$O; (**C**) Eu(TPTZ)(amcpa)$_3$·3H$_2$O; (**D**) Eu(phen)(PTA)$_{1.5}$·3H$_2$O; (**E**) Eu(TPTZ)(BA)$_3$·3H$_2$O. Samples 2~5 represent the complex added with the Ag@SiO$_2$ nanoparticles with core size of 80–100 nm and shell thickness of 5, 12, 20, 30 nm. 6~8 represent the complex added with the Ag@SiO$_2$ nanoparticles with core size of 40–60 nm and shell thickness of 12, 30 and 40 nm, respectively.

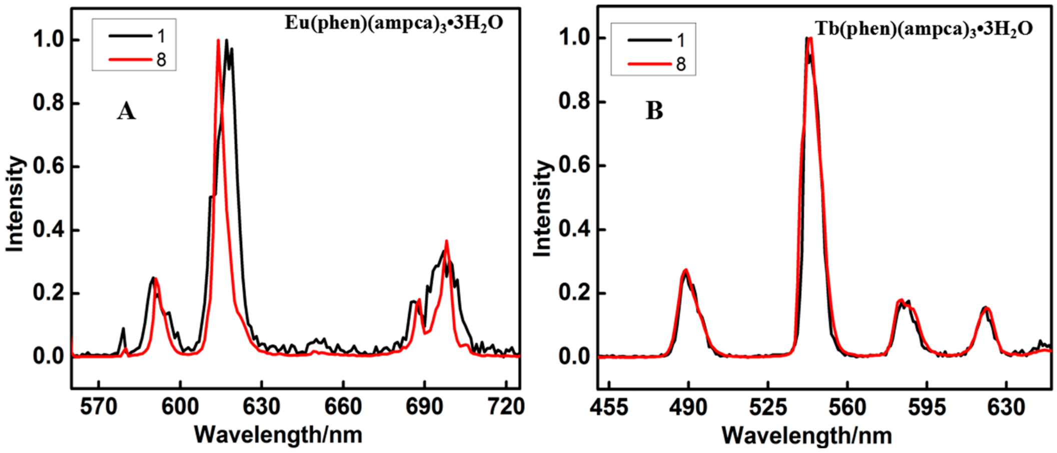

Fig S6. Normalized luminescent emission spectra of Eu(phen)(ampca)$_3$·3H$_2$O (**A**) and Tb(phen)(ampca)$_3$·3H$_2$O (**B**) before (sample 1) and after (sample 8) the addition of Ag@SiO$_2$ nanoparticles with core size of 40–60 nm and shell thickness of 40 nm.
